# Dual Biocontrol and Plant Growth-Promoting Effects of *Trichoderma nordicum* V1 Against Oomycete Plant Pathogens

**DOI:** 10.3390/jof12040292

**Published:** 2026-04-20

**Authors:** Songrong Li, Xian Wen, Siqiao Chen, Yishen Zhao, Jinhao Chen, Wanrong Li, Yajuan Chen, Mingyue Ding, Siqi Jiang, Wilfred Mabeche Anjago, Dongmei Zhou, Feng M. Cai, Irina S. Druzhinina, Min Jiu, Lihui Wei, Paul Daly

**Affiliations:** 1 College of Food and Bioengineering, Henan University of Science and Technology, Luoyang 471023, China; 230320070635@stu.haust.edu.cn (S.L.); 210321090590@stu.haust.edu.cn (X.W.); 210321090599@stu.haust.edu.cn (Y.Z.); 230320070648@stu.haust.edu.cn (J.C.); 2 Institute of Plant Protection, Jiangsu Academy of Agricultural Sciences, Nanjing 210014, China; 33315220@njau.edu.cn (S.C.); ts23040231p31@cumt.edu.cn (W.L.); ts22040220p31@cumt.edu.cn (Y.C.); 20230010@jaas.ac.cn (W.M.A.); dongmeizhou@jaas.ac.cn (D.Z.); weilihui@jaas.ac.cn (L.W.); 3 Key Lab for Organic-Based Fertilizers of China and Jiangsu Provincial Key Lab for Solid Organic Waste Utilization, Nanjing Agricultural University, Nanjing 210095, China; 2020203025@stu.njau.edu.cn (M.D.); 2019203045@njau.edu.cn (S.J.); 4 Key Laboratory of Coal Processing and Efficient Utilization, Ministry of Education, China University of Mining and Technology, Xuzhou 221116, China; 5 School of Ecology, Sun Yat-sen University, Shenzhen 518000, China; caif8@mail.sysu.edu.cn; 6 Department of Accelerated Taxonomy, The Royal Botanic Gardens Kew, London TW9 3AE, UK; i.druzhinina@kew.org

**Keywords:** pepper blight, *Trichoderma nordicum*, antagonism, biocontrol

## Abstract

The potential of *Trichoderma nordicum* (*Hypocreales*, *Ascomycota*), a recently described species, for antagonism and use in the biocontrol of oomycete-caused plant diseases is unknown. *Trichoderma* is a well-known genus for containing microbial antagonists and biocontrol agents. The *T. nordicum* in this study was isolated from decomposing wood, and *rpb2* and *tef1* barcode sequencing demonstrated that the isolates were a match to the reference *T. nordicum* and *T. nigricans* strains. Since *T. nordicum* was described before *T. nigricans*, the isolates were assigned to *T. nordicum*, although taxonomic uncertainty between these species requires future clarification. In dual-culture confrontation assays, *T. nordicum* overgrew five economically important oomycete plant pathogens (*Phytophthora capsici*, *P. sojae*, *Pythium aphanidermatum*, *P. myriotylum*, and *Globisporangium ultimum*). The inability to recover viable *P. aphanidermatum* and *P. capsici* from the parts of the plate overgrown by *T. nordicum*, coupled with protease and endo-cellulase activities, correlates with *T. nordicum* having antagonistic abilities. Inoculation with *T. nordicum* preventively reduced the levels of cucumber seedling damping-off caused by *P. aphanidermatum* by up to 70%. The *T. nordicum* biocontrol effects against pepper blight caused by *P. capsici* were greater than 80%, compared to an autoclaved *T. nordicum* spore control. *T. nordicum* could also significantly promote the growth of pepper, with plant weight increased by up to 40%, compared to an autoclaved-spore control. In contrast, *T. nordicum* could not be used to control *Pythium* soft rot of ginger caused by *P. myriotylum*, even though *P. myriotylum* was overgrown by *T. nordicum*, suggesting host- or pathosystem-specific factors influence biocontrol efficacy. In summary, *T. nordicum* is a promising biocontrol agent for use in the control of pepper blight caused by *P. capsici*, and also has potential for use in the control of other oomycete-caused plant diseases in vegetable production systems.

## 1. Introduction

Developing effective biological control is important as there are objectives to reduce or phase out certain conventional pesticides, especially in regions such as Europe, due to the potential unintended environmental damage of pesticides [1]. The *Trichoderma* genus includes important microbial antagonists, plant-growth-promoting, and plant immunity-inducing strains that have applications in the biological control of plant diseases [2]. The ancestral lifestyle of the *Trichoderma* genus is considered to be mycoparasitism [3], and *Trichoderma* genomes encode inventories of antagonism-related genes encoding such enzymes as chitinases, proteases, as well as secondary metabolite gene clusters [4]. A key part of the successful use of *Trichoderma* species in the biocontrol of crop diseases is that their use can combine microbial antagonism, parasitism, plant growth promotion, and plant immunity-inducing effects [5]. It is challenging to develop effective biocontrol of oomycete plant pathogens, and further screening and analysis of appropriate potential biocontrol agents are required [6].

Oomycete plant pathogens cause devastating diseases across diverse cropping systems, with species in the genera *Phytophthora*, *Pythium*, and *Globisporangium* responsible for billions of dollars in annual crop losses worldwide [7]. Oomycete plant pathogens are notoriously difficult to control due to their production of resilient oospores, rapid asexual reproduction via sporangia and zoospores, and broad host ranges [6]. *Phytophthora capsici* causes blight diseases on solanaceous and cucurbit crops [8], and *Pythium* and *Globisporangium* species cause seedling damping-off and root rots across numerous crop species [9]. The effectiveness of biocontrol agents against oomycetes can vary substantially depending on the specific pathogen-host-biocontrol agent combination [6], necessitating a comprehensive evaluation of biocontrol candidates against multiple pathosystems. Interestingly, oomycete cell walls contain cellulose rather than chitin [10], which requires biocontrol agents to produce cellulases in addition to, or instead of, chitinases for effective parasitism or predation. The *Trichoderma* genus is well-known for producing cellulases [11], especially *T. reesei* [12], making *Trichoderma* particularly suitable for parasitizing oomycetes [13].

Among crops infected by oomycete plant pathogens, chili pepper (*Capsicum annuum*) represents a high-value target for biocontrol deployment. Chili pepper fruits have uses as vegetables, ingredients, coloring agents, and in traditional medicine [14]. Global fresh pepper production is approximately 40 M metric tons [15] and has an economic value of approximately USD 4 billion. Although pepper faces multiple disease challenges—including anthracnose caused by *Colletotrichum* spp. [16], bacterial spot of pepper caused by *Xanthomonas* spp. [17], southern blight of pepper caused by *Sclerotium rolfsii* [18], and tomato spotted wilt virus [19]—Phytophthora blight caused by *P. capsici* is a particularly important threat [8]. *P. capsici* is a hemibiotrophic, broad-host-range oomycete plant pathogen with a global distribution and is well-known for its ability to evade disease management strategies [20,21]. Apart from *P. capsici*, other oomycete species cause economically significant diseases across diverse vegetable crops. *P. aphanidermatum* is a major cause of cucumber seedling damping-off and root rot, particularly in greenhouse production systems [22], while *P. myriotylum* causes devastating soft rot of ginger rhizomes, leading to substantial losses [23].

*P. capsici*-caused pepper blight is difficult to control, partly because of the broad-host range of *P. capsici*, the high genetic variation in *P. capsici* populations, and the persistence in soils [20]. Disease control of *P. capsici*-caused diseases includes cultural practices, water management, host resistance, conventional fungicides, and biocontrol methods [20]. There are several examples of *Trichoderma* species being used in the biocontrol of pepper blight caused by *P. capsici*. *T. brevicompactum* 6311 (sect. *Pachybasium*) inhibited *P. capsici* mycelial growth, inhibited fruit infection, and its use led to control of *P. capsici*-caused disease in a pot trial [24]. Both *T. aggressivum* f. sp. *europaeum* (*Harzianum/Virens* clade) and *T. longibrachiatum* (sect. *Longibrachiatum*) reduced the mycelial growth of *P. capsici* and their use as part of a biocontrol treatment reduced the disease levels on pepper caused by *P. capsici* [25]. *T. asperellum* strain T34 (sect. *Trichoderma*) was used for control of *P. capsici*-caused pepper disease, and this strain is the active agent in the commercial T34 Biocontrol^®^ product [26]—an excellent example of the commercial viability of *Trichoderma*-containing biocontrol methods. While some *Trichoderma* strains demonstrate broad-spectrum activity against multiple oomycetes, others show pathogen- or crop-specific efficacy, highlighting the importance of evaluating biocontrol candidates across multiple pathosystems rather than relying solely on *in vitro* antagonism assays or single-disease trials.

Recently, the *T. nordicum* (sect. *Trichoderma*) species was identified from northern China, where it had distinct barcode marker sequences compared to the related *T. atroviride* species [27]. The novelty of our study and a key knowledge gap is how the biocontrol potential of *T. nordicum* remains unexplored. We identified *T. nordicum* isolates from our own screening, and our two key aims were to determine, firstly, whether the *T. nordicum* isolates had antagonistic abilities comparable to those of other *Trichoderma* species, and, secondly, whether the *T. nordicum* isolates could be used as part of the biocontrol of multiple oomycete-caused plant diseases.

## 2. Materials and Methods

### 2.1. Isolation and Identification of Trichoderma Strains

Samples with green-colored spores were collected from the stump of a *Magnolia grandiflora* tree in Xianlin district, Nanjing, China, in May 2020, and were single-spore purified and stored as spore glycerol stocks at −80 °C.

The molecular identification of the *Trichoderma* isolates was carried out following the methods described by Cai and Druzhinina [28]. The internal transcribed spacer (ITS) region was amplified with the ITS5 (5′ GGAAGTAAAAGTCGTAACAAGG 3′) and ITS4 (5′ TCCTCCGCTTATTGATATGC 3′) primer pair [29]. The partial sequences of *tef1* were amplified with Tef1-728-F (5′ CATCGAGAAGTTCGAGAAGG 3′) and Tef1-R (5′ GCCATCCTTGGGAGATACCAGC 3′), and *rpb2* with rpb2-F (5′ CCGGCTGAGACCCCTGAAG 3′) and rpb2-R (5′ CCCATGGCTTGTTTACCCAT 3′). Note that the above *rpb2* primers were designed using the *T. atroviride rpb2* sequence because the partially degenerate primers fRpb2-5F and fRpb2-7cR [30] could not amplify successfully from the gDNA samples. The PCR was performed using standard methods, and the products were sequenced using Sanger sequencing. The ITS region, *rpb2*, and *tef1* sequences were trimmed to improve identification accuracy, according to Cai and Druzhinina [28], before using BLAST (version 2.17.0) or pairwise sequence alignment.

For phylogenetic analysis, sequences were aligned using MUSCLE [31] in MEGA12 [32], and the maximum likelihood method was used to construct the phylogenetic trees with the Tamura–Nei substitution model with a gamma distribution and invariable sites. All positions with less than 95% site coverage were excluded, and 500 bootstrap replicates were performed.

The three barcode sequences from the *T. nordicum* V1 isolate were deposited in GenBank with the accession numbers PQ489411.1 (ITS region), PQ510043.1 (*rpb2*), and PQ510044.1 (*tef1*). The *T. nordicum* isolates were deposited in the China General Microbiological Culture Collection (CGMCC) with deposit numbers CGMCC No. 41478 (*T. nordicum* V1) and CGMCC No. 41477 (*T. nordicum* V4).

### 2.2. In Vitro Assays for Antagonism Towards Oomycetes

For plate confrontation assays, as well as for the *T. nordicum* isolates, three other *Trichoderma* reference species were used for comparison: *T. asperellum* CBS433.97, *T. atroviride* IMI 206040, and *T. virens* Gv29-8. These strains were confronted with the oomycete plant pathogens *P. capsici* LT263 [33], *P. sojae* P6497 [34], *P. myriotylum* SWQ7 [35], *P. aphanidermatum* HBT1 [13], and *Globisporangium ultimum* G001 [13]. Stocks of oomycetes were maintained on 10% cV8 juice agar [36] slants at 12 °C.

For the confrontation assays, both *Trichoderma* and the oomycete species were pre-cultured on cV8 agar at 25 °C in the dark. Each 5 mm agar plug covered with hyphae from the hyphal front was transferred onto the opposite side of the 9 cm Petri dish containing 12.5 mL cV8 medium to initiate the confrontation assays (at least three replicate plates were used for each confrontation). Single-species cultures were also inoculated to determine the growth rate under the same conditions as the confrontation assay. Whether both species in the confrontation are inoculated simultaneously or at different times depends on their growth rates, with the aim of inoculating so that the colonies of the two species confront each other approximately in the middle of the plate. These cultures were incubated at 25 °C for up to 15 d under diffuse light (12 h/d). The scoring system was based on the scale of Bell [37]. On every odd-numbered day after hyphal contact in the dual culture, an antagonistic score from 1 to 5 was given based on the coverage of the *Trichoderma* colony where 1 = complete overgrowth by *Trichoderma*, 2 = partial overgrowth of the oomycete and the ⅔ coverage of Petri dish by *Trichoderma*, 3 = a type of deadlock where neither of the species overgrows each other, 4 = a partial overgrowth of *Trichoderma* and the ⅔ coverage of Petri dish by the oomycete species, and 5 = a complete overgrowth of *Trichoderma* by the oomycete species. The proportion of overgrowth was determined by the proportion of the *Trichoderma* colony that appeared to be on top of the oomycete colony, as viewed from the side of the Petri dish under back illumination at this time point. The raw scores from the confrontation are included in Table S1.

To test if the hyphae of the host/prey were still viable, mycelial plugs from various parts of the *P. aphanidermatum* and *P. capsici* colonies that were overgrown by the *T. nordicum* V1 isolate were transferred to cV8 agar and incubated at 33 °C. A temperature of 33 °C was found suitable for *P. aphanidermatum* and *P. capsici* to grow (if viable), but *T. nordicum* growth was inhibited.

### 2.3. Microscopy on In Vitro Confrontation Assays and Trypan Blue Staining

A mycelial plug of *P. capsici* LT263 or *P. myriotylum* SWQ7 was inoculated on one side of 3.5 cm plates (Nest^®^ (Wuxi NEST Biotechnology, Wuxi, China) Cat# 706001) containing cV8 agar, and *T. nordicum* V1 or V4 was inoculated on the opposite side at distances or times so that the hyphae of both species would meet in the middle of the plate. The plates were incubated at 25 °C, and after the hyphae contacted, the plates were stained with 1 mL of 0.01% trypan blue for 3 min, then rinsed with water until the background color was mostly removed. Hyphal morphology and color were imaged using a Nexcope NE900 microscope (Ningbo Yongxin Optics, Ningbo, China). The clear presence of septa in the hyphae of *T. nordicum* was used to distinguish it from the aseptate oomycete hyphae.

### 2.4. Measurement of Cellulase and Protease Activities

To measure endo-cellulase activity, mycelial plugs from the *T. nordicum* V1 isolate and *T. atroviride* IMI206040 were inoculated onto plates containing either 0.4% *w*/*v* inactivated *P. myriotylum* mycelial powder, 1% *w*/*v* cellulose (from cotton linters, Sigma, St. Louis, MO, USA, Cat# 435236), 25 mM fructose, or without an added carbon source in a minimal medium with 0.8% *w*/*v* agar described previously [13], and incubated at 25 °C. These plates contained 0.1% AZCL HE-cellulose (Megazyme, Bray, Ireland) to measure the endo-cellulase activity, where the size of the blue-dyed zone from the degradation of the AZCL-HE-cellulose was used to semi-quantify endo-cellulase activity. The *P. myriotylum* SWQ7 mycelial powder was prepared according to a previous study, where compositional analysis of the powder was also performed [13,38]. To measure protease activity, 1% *w*/*v* skim milk in 0.8% *w*/*v* water agar plates were used, and a polycarbonate membrane with a 0.1 μm pore size (GVS filter technology, Bologna, Italy, Cat# 1215304) was used to separate the colony from the skim milk agar to facilitate measurement of the clearing zone beneath. The skim milk agar plates were inoculated and incubated in the same way as the AZCL-HE-cellulose containing plates above.

### 2.5. Fungal Inoculum Preparation Methods

The solid-state fermentation medium consisted of 17.1 g wheat bran, 11.4 g rice hulls, 0.5 g ammonium sulfate, 14 g sucrose, 0.5 g potassium dihydrogen phosphate (KH_2_PO_4_), 0.57 g soybean meal powder, and 43 mL distilled water. The medium was sterilized by autoclaving and inoculated with 5.2 × 10^5^ *Trichoderma* spores per gram of medium, using spores harvested from a 6-day-old PDA plate culture, and incubated at 25 °C under light. The culture was stirred at 60 h after inoculation, and cultured statically until 8 d, when spores were harvested with sterile water.

The liquid-fermentation medium consisted of maize powder 4.5% *w*/*v*, glucose 2% *w*/*v*, yeast extract 0.5% *w*/*v*, at an initial pH 5, with or without Shengli lignite 2.5% *w*/*v* (~2 mm diameter). The source and preparation of Shengli lignite were described previously [39]. The liquid-fermentation medium (75 mL in 250 mL flasks) was inoculated with 3 mL (total of 8 × 10^8^ spores) of *T. nordicum* V1 isolate spore suspension, and shaken at 180 RPM, at 28 °C in darkness for 7 d. During the culture period, samples were initially collected every 12 h to monitor spore germination, and then, after germination and the onset of sporulation, samples were collected every 24 h to determine spore concentration using a hemocytometer. To test spore viability, the conidial suspensions collected at days 6 and 7 after inoculation were filtered through a 30 μm pore-size nylon membrane to remove hyphal fragments and plated on PDA.

### 2.6. Cucumber Seedling Damping-Off Biocontrol Assays

Cucumber (*Cucumis sativus*) “Lufeng” variety seeds were surface-sterilized by immersing in 75% ethanol for 2 min, then washed three times with sterile water, followed by 1% NaClO, and shaken at 200 RPM and 25 °C for 30 min, followed by extensive washing to remove the bleach. The air-dried cucumber seeds were sown on 1% *w*/*v* water agar, with eight seeds per plate. Seedlings were grown in a growth cabinet at 25 °C (12 h light and 12 h dark cycle). When the cucumber seedling roots reached approximately 1 cm in length, six seedlings were retained per plate. *T. nordicum* was inoculated onto the seedling in 85 μL of 10^7^ spores/mL spore suspension, and *P. aphanidermatum* HBT1 was inoculated using a mycelial plug from a cV8 culture at 1 cm below the seedling root. On the same day, seedlings were inoculated with *T. nordicum* for the treatments where *T. nordicum* was inoculated first, and seedlings were inoculated with *P. aphanidermatum* in the treatment where *T. nordicum* was inoculated second. Two days later, *P. aphanidermatum* was inoculated into treatments previously inoculated by *T. nordicum*, and *T. nordicum* was inoculated into treatments previously inoculated by *P. aphanidermatum*. Single-culture inoculated controls of *P. aphanidermatum* and *T. nordicum*, and non-inoculated controls were also included. Note that a mock inoculation of autoclaved *T. nordicum* spores was also used. Each treatment included 18 seedlings. At day 9 after the first inoculations, the disease severity of cucumber seedlings was recorded using the scale 0 = Seedlings are healthy; 1 = Seedling roots show slight brown lesions; 2 = Seedling roots show large brown lesions; and 3 = Seedlings are dead. The disease index and control efficacy were calculated from these severity scores.

### 2.7. Pepper Phytophthora Blight Disease Biocontrol Assays

Pepper (*Capsicum annuum*) cv. Sujiao No. 5 seeds were germinated in a soil:vermiculite (5:2) mixture in a growth chamber at 25 °C with 12 h of light (~5000 Lux) per day. Pepper seedlings at the 4–6 leaf stage were transplanted to 240 mL plastic pots containing 180 mL of a soil:vermiculite (5:2) mixture, and placed on an inverted Petri dish lid to prevent the spread of inoculum in the tray. The pot was covered with a larger plastic pot to maintain high humidity and prevent the spread of inoculum and cross-contamination. Pots were randomly arranged across three shelves in the growth cabinet. To minimize positional bias, pot positions were rotated each time they were removed for watering, inoculation, or assessment. Approximately 3 and 6 days after transplanting, the seedlings were fertilized with an NPK (20-20-20) fertilizer containing trace elements. On day 7 after transplanting (approximately four-leaf stage), a frozen 4 mL *T. nordicum* spore suspension, to give a final spore concentration of 1 × 10^6^/mL in the soil:vermiculite mixture, was applied around the seedling stem base and allowed to thaw and slowly drip down into the soil:vermiculite mixture. The spores from solid-state fermentation were frozen horizontally overnight at −20 °C (the purpose of freezing is to prevent too many spores from collecting at the base of the pot by slowing their passage through the soil:vermiculite mixture). A control group received 4 mL of autoclaved V1 or V4 spore suspension. One week later (approximately six-leaf and one heart stage), seedlings were inoculated ~2 cm below the stem base using a syringe with 5 mL of a *P. capsici* LT263 blended mycelium suspension or an autoclaved suspension. The *P. capsici* LT263 inoculum was prepared from a 7-day 10% cV8 shake flask culture inoculated with mycelium-covered agar plugs from the colony edge of the cV8 plate culture (~ 20 × 3 mm plugs per 300 mL cV8). The shake flask culture was incubated at 25 °C in the dark at 100 RPM for 7 d, and the pellet-form culture was blended to form a cloudy suspension. Each treatment consisted of 10 pots per replicate. The disease severity was recorded from 7 DAI to 21 DAI. Disease severity was assessed by two independent evaluators, and while complete blinding was not feasible due to visible treatment differences, the criteria were strictly followed to maintain objectivity. The disease severity rating scale was as follows: 0 = no symptoms; 1 = slight blackening of root and stem base in seedlings, leaves not wilted or showing recoverable wilting; 2 = blackening of root and stem base extending 1–2 cm, irreversible leaf wilting, occasional lower leaf abscission; 3 = blackening of root and stem base exceeding 2 cm, obvious leaf wilting or significant leaf abscission; 4 = root and stem base blackened and constricted, complete defoliation except for growing point, or plant wilting; and 5 = plant death. Disease index = Σ(number of plants at each disease grade × representative value of each grade)/(total number of plants surveyed × maximum disease grade value) × 100%. For growth promotion effects, pepper plant height, root length, plant fresh weight, and dry weight were measured 28 d after inoculation with *T. nordicum*.

For the re-isolation of *P. capsici* and *T. nordicum*, pepper root samples were collected from pots, gently washed with sterile tap water to remove soil and vermiculite, and surface-sterilized with 1% NaClO for 3 min in a small dish, and washed several times to remove the bleach. The surface-sterilized root samples were cut into ~0.5 cm pieces and placed on 10% cV8 agar (containing 100 µg/mL ampicillin) and incubated at 25 °C for 2 d. Agar plugs were subcultured from the edges of colonies that grew out from the root pieces onto another cV8 plate, and mycelia were sampled for gDNA extraction. The identity of the colonies was determined by PCR amplification of the ITS region using ITS5 and ITS4 primers, sequencing, and pairwise alignment, as described in Section 2.1.

### 2.8. Pythium Soft Rot of Ginger Disease Biocontrol Assays

The method for the ginger pot trial was based on that described previously [13]. Tissue-cultured-derived ginger seedlings (“Laiwu big ginger” variety) were transplanted into sterilized vermiculite in a growth chamber at 25 °C with 12 h of light (~5000 Lux) per day. At day 7 after transplantation, 4 mL of *T. nordicum* V1 frozen spore suspension (prepared as described in the previous section for the pepper pot trial) at final concentrations in the vermiculite of 1 × 10^5^ spores/mL and 1 × 10^6^ spores/mL were inoculated into the vermiculite around each ginger seedling. The control group received 4 mL of heat-inactivated (autoclaved) V1 spore suspension. To prepare the *P. myriotylum* inoculum, wheat seeds were soaked in distilled water for 24 h, then autoclaved twice and spread evenly on plates containing *P. myriotylum* SWQ7, and cultured for a further ~5 d. Four mycelium-covered wheat seeds were inoculated into the vermiculite around the roots of each ginger plant. Photos were taken, and disease severity was recorded 15 DAI of *P. myriotylum* SWQ7. Each treatment included 12 ginger seedlings, and the experiment was repeated two times. Disease severity rating scale: 0 = healthy ginger seedling, 1 = yellowing of lower leaves, 2 = all leaves yellowed but plant not dead, and 3 = plant dead. The disease index was calculated as: Disease index = Σ(number of plants at each disease grade × representative value of each grade)/(total number of plants surveyed × maximum disease grade value) × 100%. For growth promotion effects, ginger plant fresh weight and dry weight were measured 21 d after inoculation with *T. nordicum*. For the re-isolation of *P. myriotylum* and *T. nordicum*, ginger root samples were collected from pots following the same procedure as described for the pepper pot trial in Section 2.7.

### 2.9. Statistical Analysis

Statistical analysis was carried out using GraphPad Prism 9.5.0 (GraphPad Software, San Diego, CA, USA). The Kruskal–Wallis non-parametric test and Dunn’s post hoc test (this post hoc test was chosen because it can compare treatment ranks to the rank values of a single control after a non-parametric test) were used for disease indices (disease indices are ordinal and non-normally distributed and thus require a non-parametric test). ANOVA with Dunnett’s post hoc test (this post hoc test was chosen because it can compare treatment means to a single control after a parametric test) was used for growth-related measurements. Student’s *t*-tests with an FDR correction were used for spore yields and viability measurements. For the calculation of AUDPC, the area-under-the-curve function in GraphPad Prism was used, which uses the trapezoidal rule.

## 3. Results

### 3.1. Identification of Trichoderma Isolates as Belonging to the T. nordicum/T. nigricans Species Complex

Isolates were collected from the stump of a *Magnolia grandiflora* tree in Nanjing, China, and two of the isolates were identified as *T. nordicum*/*T. nigricans* species. The ITS region sequences of the V1 and V4 isolates were identical to each other (GenBank accession: PQ489411.1) and were a match of 100% identity to species from the *Trichoderma* genus in GenBank, and therefore met the initial criteria to continue with more accurate identification of the strain using *rpb2* and *tef1* sequences. The trimmed V1 and V4 *rpb2* sequences were identical to each other (GenBank accession: PQ510043.1) and were identical to the *rpb2* sequence (MH287502.1) of the *T. nordicum* type strain ACCC 39713 (WT13001) [27] and the *rpb2* (OP357959) of the *T. nigricans* type strain T32781 = CGMCC40314 [40]. The trimmed V1 and V4 *tef1* sequences were also identical to each other (GenBank accession: PQ510044.1) and had a similarity of 99% with the *tef1* sequence (MH287501.1) of the same *T. nordicum* type strain and 99% with the *tef1* sequence (OP357974) of the same *T. nigricans* type strain (Table 1). Importantly, the V1 and V4 sequences had lower similarity with the *T. atroviride* reference strain CBS142.95 sequences, supporting that V1 and V4 were *T. nordicum* or *T. nigricans* isolates and not *T. atroviride*. Since *T. nordicum* was described earlier [27] than *T. nigricans* [40], the *T. nordicum* species was assigned to the V1 and V4 isolates. See the Discussion section regarding a discussion of the taxonomic assignment of the V1 and V4 isolates, and how there is uncertainty in the assignment of *T. nordicum* compared to *T. nigricans*.

**Figure 1:**
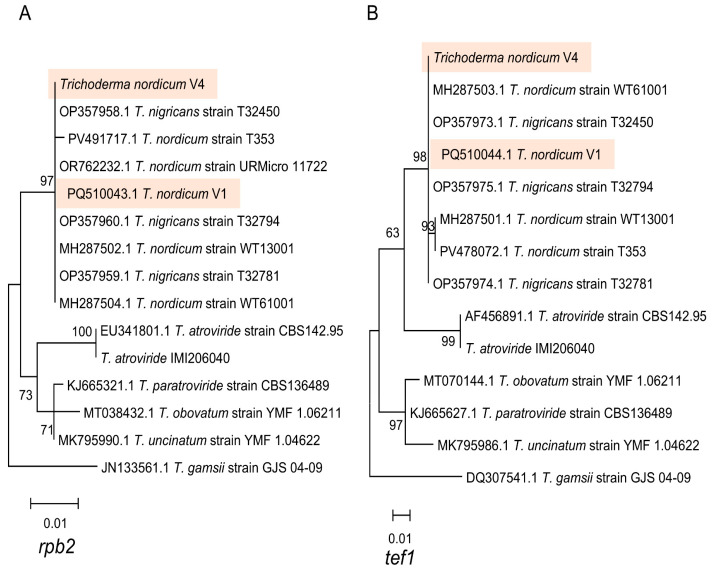
Maximum likelihood phylogenetic trees for (**A**) *rpb2* and (**B**) *tef1* showing the clustering of the *T. nordicum* V1 and V4 isolates (highlighted with the colored boxes) with the type strain *T. nordicum* WT13001 and the type strain *T. nigricans* T32781. Bootstrap values > 60% are shown on trees. There were 566 positions in the alignment for the *rpb2* tree, and there were 262 positions in the alignment for the *tef1* tree. Note that the *T. atroviride* IMI206040 sequences were obtained from the genome sequence of the strain.

The phylogenetic trees constructed based on the *tef1* and *rpb2* genes are shown in Figure 1, and there was high bootstrap support for the clustering of the *T. nordicum* V1 and V4 *tef1* and *rpb2* sequences with the sequences from the *T. nordicum* type strain ACCC 39713 (WT13001) and *T. nigricans* type strain T32781 = CGMCC40314, and clustering separately from the *T. atroviride* reference strain CBS142.95.

### 3.2. T. nordicum Isolates Overgrow a Range of Oomycete Plant Pathogens and Have Antagonistic Abilities

**Figure 2:**
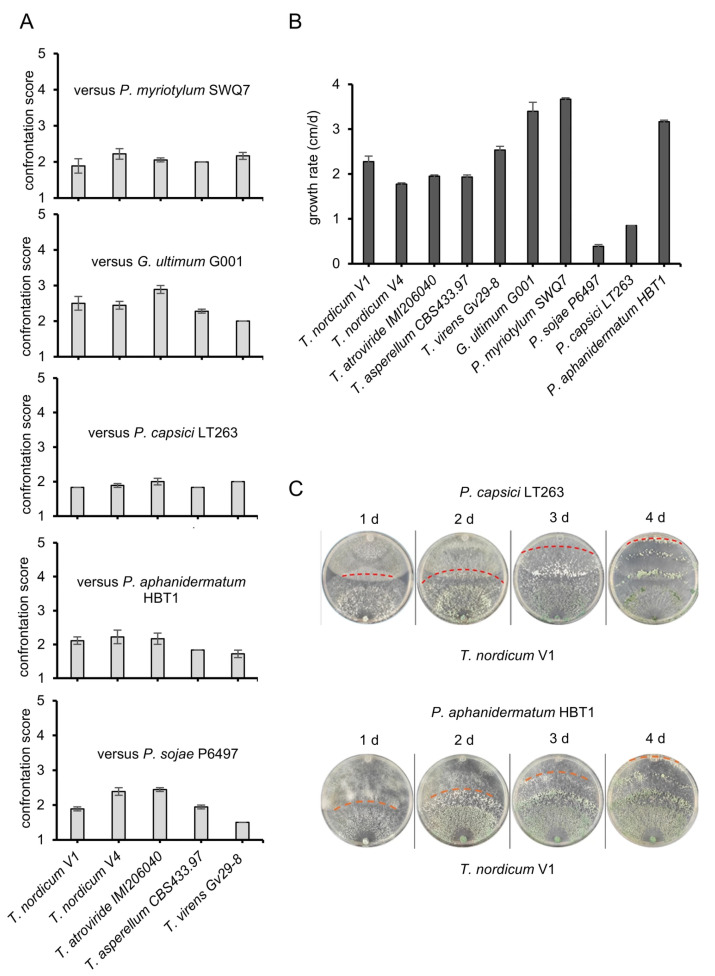
(**A**) Confrontation score from Bell’s scale (1 = the strongest level of *Trichoderma* antagonism) from the in vitro confrontation assay of *Trichoderma* strains with five oomycete plant pathogens. The score is the average of the scores given to three time points after colonial contact. (**B**) Growth rates of single cultures of *Trichoderma* and oomycete strains. (**C**) Representative images of the confrontation from days 1 to 4 after contact between *T. nordicum* and *P. capsici*, and *T. nordicum* and *P. aphanidermatum*. The dotted line marks the extent of the *T. nordicum* overgrowth. The error bars represent standard errors (*n* (plate) = 3). The raw scores from the confrontation are included in Table S1.

The *T. nordicum* and *T. nigricans* species were only recently described, and since there were no reports of antagonism by these species, the antagonism of the V1 and V4 isolates towards oomycete plant pathogens and their ability to be used in the control of oomycete-caused diseases were investigated. The ability of the *T. nordicum* isolates to overgrow two *Phytophthora* (*P. capsici* and *P. sojae*) and two *Pythium* (*P. myriotylum* and *P. aphanidermatum*) plant pathogens, and *G. ultimum* was tested. Both *T. nordicum* isolates overgrew all five of the tested plant pathogens (Figure 2A). The overgrowth of *G. ultimum* by both *T. nordicum* isolates was generally less than the overgrowth of the other plant pathogens, while the overgrowth of *P. capsici* by both *T. nordicum* isolates tended to be the most. The relatively faster growth rate of *G. ultimum* may partly explain the weaker overgrowth by the *T. nordicum* isolates (Figure 2B). Generally, the *T. nordicum* V1 isolate tended to overgrow the plant pathogens more than the V4 isolate, and in subsequent assays in which only a single isolate was used, preference was given to the V1 isolate. The *T. nordicum* isolates had comparable levels of overgrowth to those of reference strains of *T. atroviride*, *T. virens*, and *T. asperellum* (Figure 2). The raw data of the lengths of colonies, alongside the dates when those lengths were recorded (which shows the time taken to overgrow the host), and corresponding scores from Bell’s scale for individual replicate confrontations, are shown in Table S1.

**Figure 3:**
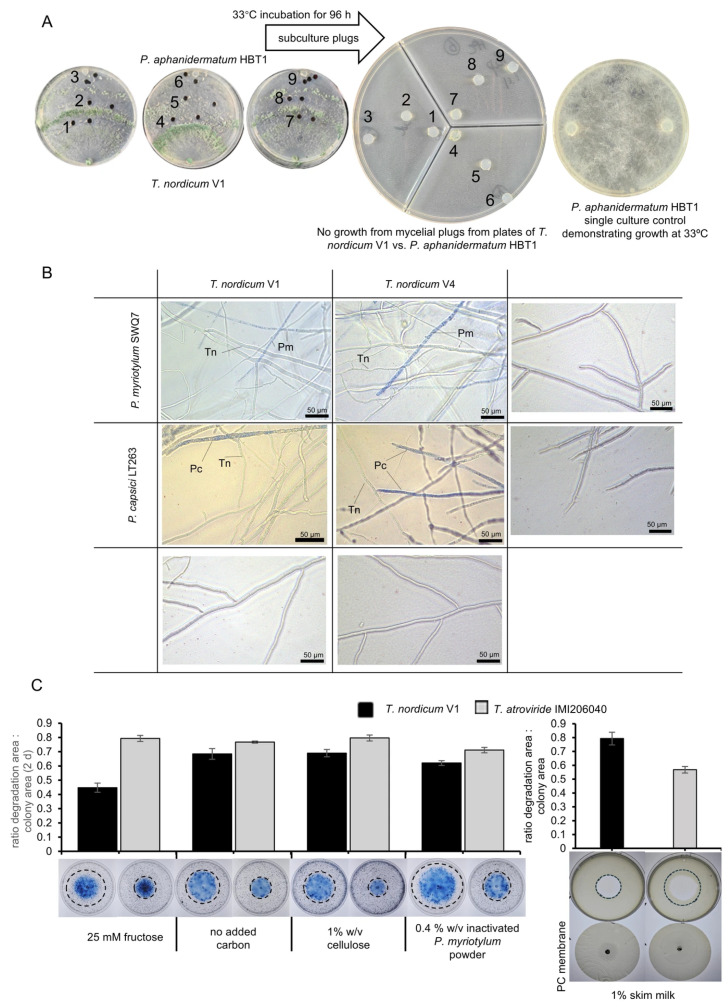
Data supporting the antagonistic ability of *T. nordicum*. (**A**) Test of viability of *P. aphanidermatum* HBT1 mycelia from parts overgrown by *T. nordicum* V1 isolate (numbers 1–9 correspond to the plugs transferred from the confrontation plate to the plate to test for plant pathogen viability); (**B**) brightfield microscopy of trypan blue-stained hyphae of confrontation between *T. nordicum* isolates and either *P. myriotylum* SWQ7 or *P. capsici* LT263; and (**C**) endo-cellulase and caseinase activity of *T. nordicum* V1 isolate and *T. atroviride* IMI206040. The dotted lines mark the edge of the colony. The error bars represent standard errors (*n* (plate) = 4).

The overgrowth by the *T. nordicum* isolates of the *Pythium*, *Phytophthora*, and *Globisporangium* plant pathogens can be due to various factors, such as competition for nutrients, predation, or parasitism. To test whether the plant pathogen was still viable when overgrown by the *T. nordicum* isolates, the ability of *P. aphanidermatum* and *P. capsici* to grow at higher temperatures than the *T. nordicum* isolates was taken advantage of. The lack of growth at 33 °C of mycelium-covered plugs taken from parts of the plate where *T. nordicum* had overgrown *P. aphanidermatum* indicated that *P. aphanidermatum* was dead in the parts of the plate where it was overgrown by *T. nordicum* (Figure 3A). Control viable mycelium plugs of *P. aphanidermatum* grew readily at 33 °C. There was a similar trend using mycelium-covered plugs from parts of the plate where *T. nordicum* had overgrown *P. capsici*, whereby no *P. capsici* growth was observed, although the growth was more difficult to image because of the slower growth rate of *P. capsici* at 33 °C (Figure S1). The lack of viability of the mycelia of *P. aphanidermatum* and *P. capsici* from the confrontation supported an antagonistic mechanism from *T. nordicum*. In the microscopy analysis of hyphae in the confrontation zone between *T. nordicum* and *P. capsici* or *P. myriotylum*, the clear presence of septa in the hyphae of *T. nordicum* was used to distinguish it from the aseptate oomycete hyphae (Figure 3B). For both *P. capsici* and *P. myriotylum* hyphae stained with the viability stain trypan blue, there were examples of blue-stained hyphae indicating a loss of viability (Figure 3B). There were no clear examples of coiling of *T. nordicum* hyphae around the hyphae of *P. myriotylum* or *P. capsici*, although it was difficult to observe the confrontation other than at initial contact points, and reliably distinguish *T. nordicum* from oomycete hyphae. Hyphal coiling is evidence supporting a parasitic mechanism, and the lack of observation of hyphal coils, as well as the lack of other direct evidence of parasitism, cautions against interpreting the loss of plant pathogen viability after *T. nordicum* overgrows as parasitism, when other mechanisms, such as antibiosis, could be responsible.

Enzymatic activities that can contribute to *Trichoderma* antagonism of oomycetes include proteases and cellulases, as reviewed previously [41]. Protease activity, measured by the hydrolysis of skim milk agar, was detected for *T. nordicum* and had similar levels of activity to the *T. atroviride* IMI206040 strain. Endo-cellulase activity, measured using dyed AZCL-cellulose, was detected for *T. nordicum* in medium supplemented with cellulose and also in medium supplemented with inactivated oomycete mycelial powder, and the cellulase activity levels were similar in the *T. atroviride* IMI206040 strain (Figure 3C). The endo-cellulase activity was also detected from *T. nordicum* and *T. atroviride* in medium supplemented with fructose and in medium without an added carbon source, suggesting that both strains have a constitutive level of endo-cellulase activity and may also use the agar as a carbon source. Considering this, the endo-cellulase activity detected from *T. nordicum* cultured on the medium with inactivated mycelial powder or cellulose may not necessarily be due to induction by these carbon sources.

### 3.3. Preventative but Not Curative Inoculation of T. nordicum Reduced Cucumber Seedling Damping-off Compared to an Autoclaved-Spore Control

**Figure 4:**
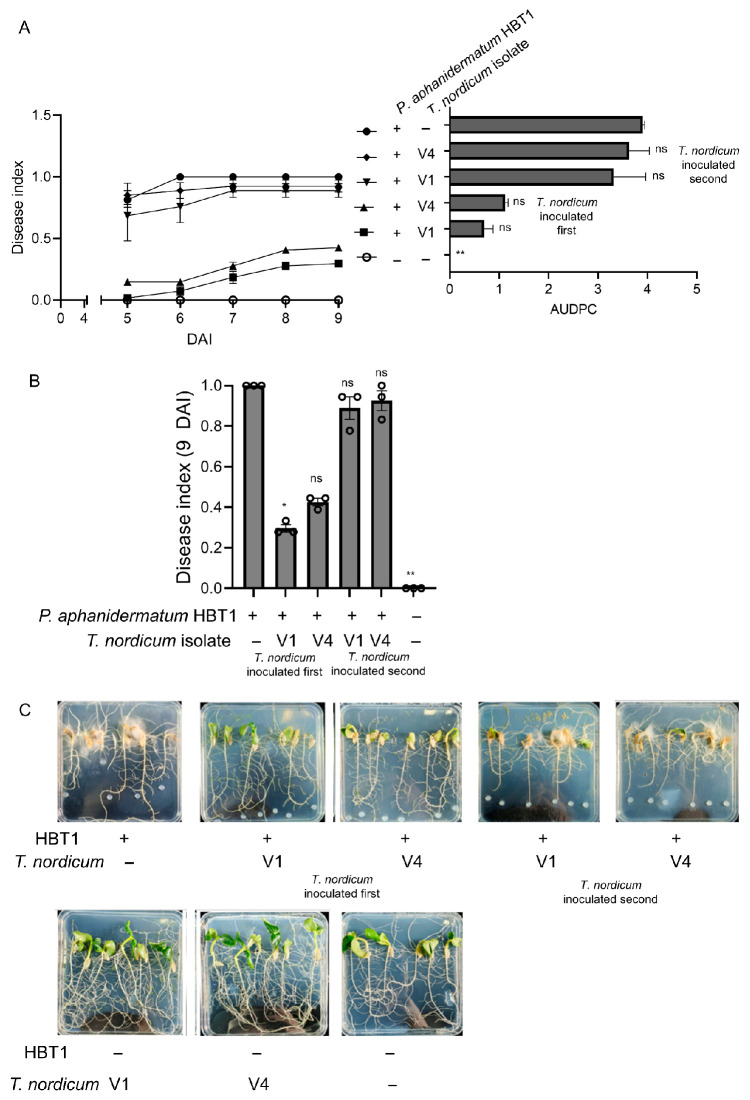
(**A**) Time course of disease index and AUDPC, and (**B**) disease index from 9 DAI from control of cucumber seedling damping-off caused by *P. aphanidermatum* HBT1 using spore inoculum of *T. nordicum* V1 and V4 isolates. The error bars represent standard errors (*n* (plates) = 3 (each plate contained six seedlings)). ** = *p* < 0.01, * = *p* < 0.05, ns = non-significant from Dunn’s post hoc test after Kruskal–Wallis test in comparison to the disease index or AUDPC of seedlings inoculated with *P. aphanidermatum* HBT1 and autoclaved *T. nordicum* spores. (**C**) Representative images from different treatments. Note that the images include two treatments not included in the graphs because only a single plate was tested to demonstrate that *T. nordicum* alone does not cause disease symptoms.

Compared to the control group inoculated with autoclaved *T. nordicum* spores followed by *P. aphanidermatum* HBT1, when V1 isolate was inoculated first (preventative inoculation), followed by *P. aphanidermatum*, the V1 isolate significantly reduced the seedling damping-off on cucumber seedlings, with significant biocontrol efficacy of 70% (*p* < 0.05, Dunn’s test following Kruskal–Wallis test) (Figure 4). There was also a substantial biocontrol effect of the V4 isolate when inoculated first, but it was not statistically significant using a non-parametric statistical method in which the plate, not individual seedlings, was considered the unit of replication (*n* = 3), which likely reduced statistical power. However, when *P. aphanidermatum* was inoculated first, followed by V1 or V4 isolates (curative inoculation), the biocontrol effects were much weaker, with neither biocontrol efficacy statistically significant. These results indicate that when using *T. nordicum* to control cucumber seedling damping-off caused by *P. aphanidermatum*, it is necessary to apply it as a preventative treatment so that *T. nordicum* can establish a competitive advantage for significant biocontrol efficacy.

### 3.4. Greater than 80% Biocontrol of Pepper Blight Disease Caused by P. capsici Using T. nordicum Compared to an Autoclaved-Spore Control

**Figure 5:**
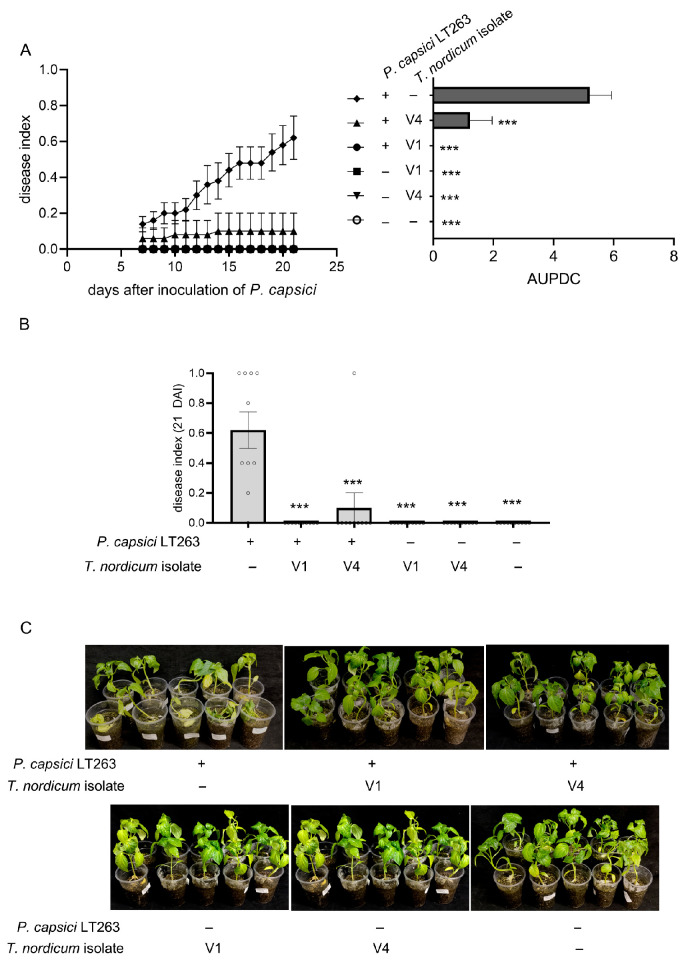
(**A**) Time course of disease index and AUDPC and (**B**) disease index from 21 DAI from control of *T. nordicum* on pepper blight caused by *P. capsici*. The error bars represent standard errors (*n* (plants) = 10). *** = *p* < 0.001 from Dunn’s post hoc test after Kruskal–Wallis test in comparison to the disease index or AUDPC of seedlings inoculated with *P. capsici* LT263 and autoclaved *T. nordicum* spores. (**C**) Images of pepper plants 21 d after inoculation of *P. capsici*. The pot trial was repeated three times with similar results (see Figure S2 for the results of the other two repeats).

With the ability of *T. nordicum* to antagonize *P. capsici* established in the confrontation assays (Figure 2), the use of *T. nordicum* for the biocontrol of Phytophthora pepper blight caused by *P. capsici* was tested. *T. nordicum* spores were inoculated onto a subset of the pepper seedlings, followed by inoculation with *P. capsici* 7 d later. In the pepper plants inoculated with *P. capsici* and inactivated (autoclaved) spores of *T. nordicum*, there were clear signs of symptoms associated with Phytophthora-caused pepper blight, such as wilting leaves, yellowing leaves, wilting stems, and a blackening appearance of roots and blackening at the base of the stem, and the disease index was 0.62 (Figure 5). In contrast, in the pepper plants that were inoculated with *T. nordicum*, there were a few signs of pepper blight disease symptoms from the pepper plants inoculated with V4 isolate (biocontrol rate of 84%), and no symptoms were observed on pepper plants inoculated with V1 isolate (biocontrol rate of 100%). At the end of the trial, *P. capsici* was re-isolated from infected plants, and *T. nordicum* was re-isolated from healthy plants that had been inoculated with both *P. capsici* and *T. nordicum* (Figure S3).

In contrast to the use of *T. nordicum* for biocontrol of pepper blight caused by *P. capsici*, there was no clear control of Pythium soft rot of ginger caused by *P. myriotylum* compared to an autoclaved *T. nordicum* spore control (Figure S5). The above-ground disease symptoms included yellowing of ginger leaves, and there were many dead and dying ginger plants at 16 DAI of *P. myriotylum* across all treatments (Figure S5). There were no significant reductions (*p* > 0.05, Dunn’s test following Kruskal–Wallis test) in disease severity from the *T. nordicum* spore treatments on Pythium soft rot disease of ginger here. At the end of the trial, *P. myriotylum* was easily re-isolated from infected plants, and while *T. nordicum* was re-isolated from infected plants inoculated with *T. nordicum* before inoculation with *P. myriotylum*, it was easier to re-isolate *P. myriotylum* from these infected plants (Figure S6).

### 3.5. T. nordicum Promoted the Growth of Pepper by More than 40% Compared to an Autoclaved-Spore Control

*Trichoderma* is known to function via microbial antagonism-mediated mechanisms, as well as via plant-mediated mechanisms. The ability of *T. nordicum* to promote the growth of pepper plants was investigated.

**Figure 6:**
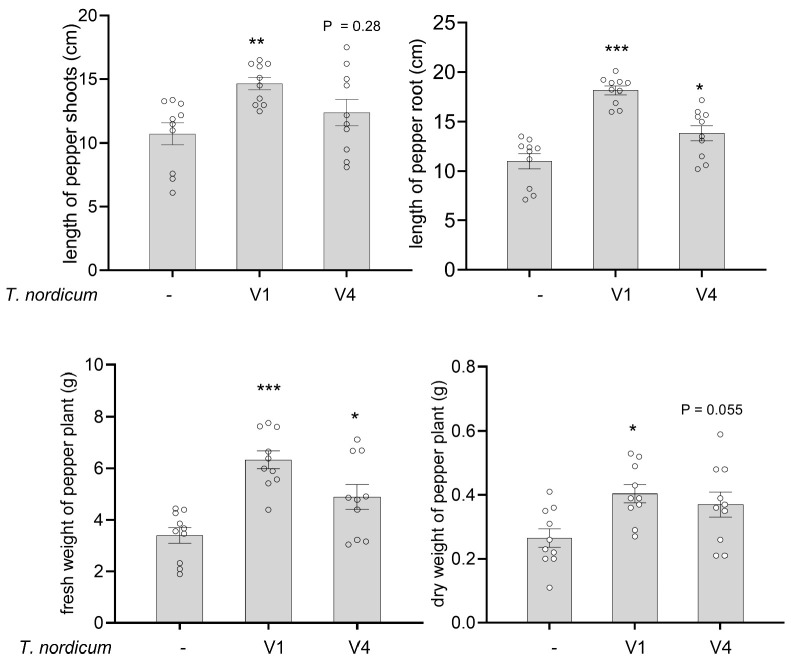
Growth promotion effect of *T. nordicum* isolates on pepper 28 d after inoculation with *T. nordicum*. The error bars represent standard errors (*n* (plants) = 10). *** = *p* < 0.001, ** = *p* < 0.01, * = *p* < 0.05 from Dunnett’s post hoc test (after ANOVA) in comparison to the seedlings inoculated with autoclaved *T. nordicum* spores (-). The pot trial was repeated three times with similar results (see Figure S4 for the results of the other two repeats).

The pot trial with pepper showed that inoculation with *T. nordicum* V1 and V4 isolates had significant growth-promoting effects on pepper compared to an autoclaved-spore control. At 4 weeks after V1 inoculation, significant increases (*p* < 0.05, Dunnett’s test following ANOVA) in plant height (37%), root length (65%), fresh weight (87%), and dry weight (53%) were measured compared to the control of autoclaved *T. nordicum* spores (Figure 6). The *T. nordicum* V4 isolate also promoted the growth of pepper plants, but to a lesser level than the V1 isolate. At 4 weeks after V4 inoculation, there were significant increases (*p* < 0.05, Dunnett’s test following ANOVA) in root length (26%) and fresh weight (44%) compared to the control, whereas there was no significant difference in plant height or dry weight (*p* > 0.05) (Figure 6). In two independent repeat experiments, there were similar trends, although there were fewer statistically significant differences, e.g., the increase in dry weight of pepper plants inoculated with *T. nordicum* V4 was significant in one of the repeats but marginally non-significant in the other repeat (*p* = 0.057, Dunnett’s test following ANOVA) (Figure S4). Of note, re-isolation of *T. nordicum* was not attempted from pepper plants from the growth promotion experiment, but it was done from roots inoculated with *T. nordicum* followed by the plant pathogen *P. capsici*, and here, *T. nordicum* was readily recovered (Figure S3), supporting that *T. nordicum* can colonize pepper roots, which is likely a factor contributing to the growth promotion effect.

In contrast, there was no clear growth promotion effect on ginger due to inoculation with *T. nordicum* V1, whereby there were no significant differences (*p* > 0.05, Dunnett’s test following ANOVA) in fresh weight or dry weight of the ginger plants inoculated to give a final spore concentration in vermiculite of either 1 × 10^5^/mL or 1 × 10^6^/mL (Figure S5).

### 3.6. Culturing T. nordicum with Lignite Increases Spore Yield

**Figure 7:**
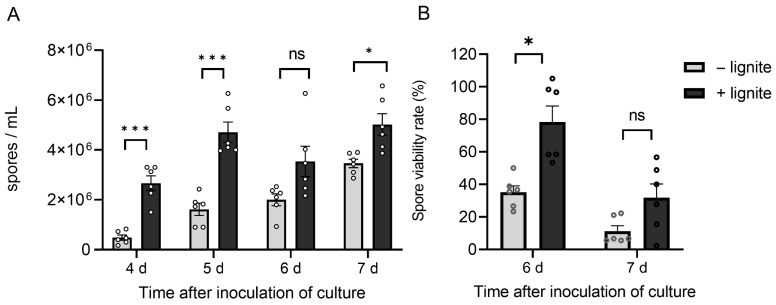
Increase in *T. nordicum* V1 spore yield in cultures supplemented with lignite. (**A**) Spore counts and (**B**) spore viability of spores cultured with and without lignite in shake flasks in liquid fermentation medium. The error bars represent standard error (*n* (flasks) = 6). *** = q < 0.0001, * = q < 0.01, ns = non-significant from multiple *t*-tests with an FDR threshold of 1%. The experiment was repeated twice with similar trends.

In the previous pot-trial experiments, the *T. nordicum* spore inoculum came from a solid-state type fermentation. Another liquid fermentation method was also tested, partly to assess the effect of lignite addition to the liquid fermentation medium. Lignite can function as a suitable carrier for fungal spores in biocontrol applications. After inoculating the media with V1 spores, samples were taken every 24 h to initially observe spore germination and later count sporulation levels. Spores began to germinate after 24 h, and almost all spores had germinated by 72 h, with some hyphae differentiating into conidiophores and beginning sporulation. On days 4, 5, and 7, the spore concentrations in the medium supplemented with lignite were ~5-fold, ~3-fold, and ~1.5-fold significantly (q < 0.01, multiple *t*-tests with an FDR threshold of 1%) higher, respectively, than the medium without lignite (Figure 7A). These spore concentrations indicated that the addition of lignite significantly increased *T. nordicum* V1 sporulation levels. The germination rate of the spores from these cultures was determined to confirm that there were no adverse effects on the spore germination rate that would obviate the benefit of higher spore concentrations. From day 6, the germination rate of spores from the medium supplemented with lignite was 78.3 ± 9.8%, which was significantly higher (q < 0.01, multiple *t*-tests with an FDR threshold of 1%) than the germination rate of spores from the medium without lignite at 35.2 ± 3.9%. This increased germination rate was also measured at day 7, when it was 31.9 ± 8.4%, which was marginally non-significant (q > 0.01) than the control group at 11.3 ± 3.3% (Figure 7B). In both days where germination rate was measured, it was clearly not significantly lower from the medium supplemented with lignite, indicating that the increased spore concentrations in the lignite-supplemented medium will potentially be beneficial for biocontrol application of this spore inoculum to plants.

## 4. Discussion

Here, we show that isolates of *T. nordicum* can antagonize oomycete plant pathogens and control Phytophthora blight of pepper caused by *P. capsici*. The efficient disease control of pepper blight is likely due to antagonism of *P. capsici*, as well as stimulation of pepper growth. Overgrowth of the plant pathogen in a plate confrontation assay did not necessarily correlate with control of disease, because while *T. nordicum* could control pepper blight caused by *P. capsici*, and cucumber seedling damping-off caused by *P. aphanidermatum*, it could not control Pythium soft rot disease caused by *P. myriotylum* (even though *P. myriotylum* was overgrown by *T. nordicum*).

The Phytophthora blight of pepper disease biocontrol was greater than 80% and comparable to or better than the highest disease control levels reported in other studies using *Trichoderma*. The use of *T. harzianum* 2413 (*Harzianum/Virens* clade) led to a reduction in pepper root rot caused by *P. capsici* by approximately 60–80%, depending on the inoculum size of *P. capsici* that was inoculated at the same time as *T. harzianum* [42]. *T. asperellum* strain T34 (sect. *Trichoderma*) led to a reduction in *P. capsici*-caused pepper disease by up to 71% in growth chamber trials where *T. asperellum* T34 was applied before *P. capsici* inoculation and again at the appearance of disease symptoms [26]. Both *T. aggressivum* f. sp. *europaeum* (*Harzianum/Virens* clade) and *T. longibrachiatum* (sect. *Longibrachiatum*) (applied before *P. capsici* inoculation) led to a reduction in the disease levels on pepper caused by *P. capsici* in a greenhouse, whereby 60% of plants were without symptoms in the *T. aggressivum* f. sp*. europaeum* treatment and 80% in the *T. longibrachiatum* treatment [25]. *T. brevicompactum* 6311 spore treatment applied before *P. capsici* inoculation was used to partially biocontrol *P. capsici*-caused disease in a pot trial, whereby the disease level was halved [24]. Several factors may explain differences in control rates compared with other studies involving *Trichoderma*, and comparisons are complicated by different growth conditions, disease scoring systems, and *P. capsici* strains and pepper varieties. For *Trichoderma*, most studies described here used a preventive method in which the biocontrol agent is inoculated before the plant pathogen. In the future, other strains/isolates of *P. capsici*, alongside other pepper varieties, could be used to test whether similar control effects from *T. nordicum* are achieved.

The results with the *T. nordicum* strains suggest several mechanisms that contribute to its use in a biocontrol treatment of pepper blight. Firstly, *T. nordicum* overgrows *P. capsici* in plate confrontation assays, and *P. capsici* was no longer viable in parts of the plate where it was overgrown by *T. nordicum*, demonstrating an antagonistic effect. Of note, the plate confrontation assays do not distinguish mycelial contact-dependent effects from those independent of contact, such as volatiles or compounds secreted into the agar medium. In the future, other plate assays could tease apart these effects, such as by using bi-partite plates to test the effects of volatiles and culturing the plant pathogens on cell-free compounds secreted by *T. nordicum*. This antagonistic effect may also be occurring in the pepper root rhizosphere, whereby *T. nordicum* antagonizes the *P. capsici* that is inoculated onto the pepper after the inoculation of *T. nordicum*. The protease and cellulase activities detected from *T. nordicum* are likely contributing to this antagonism in the plate confrontation assays. The *T. nordicum* endo-cellulase activity appears largely constitutive, as there were similar levels of endo-cellulase activity on all the tested medium supplementations, and therefore, if constitutive, there is a greater likelihood of endo-cellulase activity being present in the plate confrontation and pepper rhizosphere than an activity that would require the presence of an inducer. Previously, it was shown that cellulases were contributing to the antagonism from *T. reesei* towards *Pythium* plant pathogens [13], and *T. harzianum* strains with higher protease activity were shown to have higher antagonism levels in plate confrontation assays [43]. Secondly, there was a clear effect on pepper growth promotion after inoculation with *T. nordicum* compared to an autoclaved-spore control. This increased growth may lead to more robust plants, which are less susceptible to *P. capsici* and, in that way, also contribute to the pepper disease control effect of *T. nordicum*. The growth-promoting effects of *Trichoderma* on pepper have been reported previously, such as by *T. harzianum* and *T. asperellum* [44]. Thirdly, the control effect has a level of pathosystem-dependent performance involved in it because *T. nordicum* did not control soft rot disease of ginger caused by *P. myriotylum*, even though *T. nordicum* also overgrew *P. myriotylum* in the plate confrontation assays. Previously, another *Trichoderma* species, *T. asperellum* CBS433.97 (which also overgrew *P. myriotylum* in the plate confrontation assays), was used to partially biocontrol soft rot disease of ginger caused by *P. myriotylum*, where there was a significant control rate of ~50% [13]. Differences in *T. nordicum* biocontrol efficacy across pathosystems do not necessarily reflect plant-pathogen-specific antagonism but may result from pathosystem-dependent factors, including plant-microbe interactions and other environmental conditions that vary among the ginger, pepper, and cucumber systems tested. For example, the ginger rhizosphere may be less favorable to *T. nordicum* colonization than the pepper rhizosphere, or *P. myriotylum* genes involved in antagonism during plate confrontation may be differentially expressed in the ginger rhizosphere. Also, biochemical conditions in the ginger rhizosphere, such as pH, may be less favorable for the activity of enzymes or other metabolites that *T. nordicum* may use to antagonize *P. myriotylum* in plate culture. Of note with regard to the use of autoclaved spore inoculum as the control, it is possible that the inactivated spore suspension also has some growth-promoting effects (e.g., some polymers from spores, such as chitin, may have a type of MAMP or immune inducing function or the nutrients from the medium may alter microbiota), and in future work, it would be beneficial to compare with other controls such as water or the medium used for culturing *T. nordicum*.

The isolation source for the *T. nordicum* isolates was wood from a tree stump, which contrasts with more commonly used isolation sources for biocontrol, such as the rhizosphere of healthy plants in an area with many diseased plants [45]. On dead wood, *T. nordicum* may still function as a microbial antagonist through parasitism of other wood-associated microorganisms rather than being a primary wood decomposer. This is supported by how *Trichoderma* species can be used in the control of wood-decaying fungi in urban trees, whereby *Trichoderma* protects rather than decomposes the wood [46]. Although *T. nordicum* may not be a primary wood decomposer, its isolation from a woody substrate may mean it is tolerant to inhibitory compounds that can be found in wood, and this might contribute to its ability to be cultured with lignite, which contains phenolic and other aromatic compounds that may overlap with inhibitory compounds found in wood lignin [47]. Also, the higher *T. nordicum* spore yield in shake flask cultures with lignite may be related to the lignite pieces providing more solid surfaces for sporulation and to increased oxygen transfer due to the enhanced mixing and potential bubble formation caused by the movement of the lignite pieces. Lignite is a humic-rich substance, and this can promote plant growth, which is an additional benefit, alongside increasing *T. nordicum* spore yield, of using lignite. The potential of *T. nordicum* V1 to degrade lignite in an agricultural environment is worthwhile testing, as it may function as a carbon source as well as a carrier, and beneficially contribute to the release of humic acids from the lignite, but perhaps, adversely, it could reduce bioformulation shelf-life if the lignite can be degraded during storage. For use as a bioformulation, it may require separating the mycelial fragments from the spores and lignite pieces. Testing with smaller lignite pieces than the ~2 mm size used may be beneficial for downstream separation from the larger mycelial fragments. From a regulatory perspective, the use of lignite may require separate approval applications beyond those required for *T. nordicum* itself, especially as lignite may contain levels of metals or aromatic compounds that are environmentally toxic.

Initially, analysis of ITS region sequences indicated that the V1 and V4 isolates could be identified as *T. atroviride* or *T*. cf. *atroviride*, but further analysis indicated that the best species identification was to two species (*T. nordicum* and *T. nigricans*). Since *T. nordicum* was described earlier by [27] than *T. nigricans* (type strain: T32781 = CGMCC40314) by [40], the *T. nordicum* description, rather than *T. nigricans*, was used for the V1 and V4 isolates. In the future, further taxonomic analysis (e.g., whole genome sequencing and morphological analysis) is required to delineate the species boundaries of the *T. nordicum*/*T. nigricans* species complex, and for now, we consider V1 and V4 isolates part of the *T. nordicum*/*T. nigricans* species complex and adopt the name *T. nordicum* for the V1 and V4 isolates. Notably, a genome sequence is available for *T. nigricans* (Zhang, Qiu et al. 2025 [48]), but none is available yet for *T. nordicum*. While there are no reports in the literature of *T. nordicum* antagonizing plant pathogens, there are sequence data in GenBank from at least two *T. nordicum* strains that may be involved in this. *T. nordicum* strain URMicro 11722 (GenBank: OR762232.1) is from an entry titled about novel species of *Trichoderma* antagonistic to sclerotia-producing plant pathogens, and *T. nordicum* isolate T353 (GenBank: PV491717.1) from an entry titled identification of *Trichoderma* spp. from ancient *Platycladus orientalis* (a coniferous cypress tree) and their biocontrol for Alternaria leaf blight. There are no reports in the literature of *T. nigricans* antagonizing plant pathogens or being used as a biocontrol treatment of crop diseases. *T. nigricans* T32781 is tolerant to the metal cadmium, which can lead to reduced levels of cadmium availability and contamination in soils colonized by *T. nigricans* T32781 [48].

Future work with *T. nordicum* could involve strain engineering, chlamydospore production, testing spore carriers, and analyzing the control potential for other oomycete-caused diseases. Genome sequencing and gene expression analysis could be used in the future to identify key genetic factors for the biocontrol of *T. nordicum* strains and to engineer *T. nordicum* for improved biocontrol. Conidia from the *T. nordicum* strains were used here, but in other studies, such as with *T. longibrachiatum*, it was found that chlamydospores had greater survival ability and biocontrol potential than conidia [49], and the potential for future chlamydospore production has implications for field applications and product shelf life. The ability of the *T. nordicum* strains to be cultured with lignite also leads to the potential to use lignite as a carrier for *T. nordicum* spores, as lignite can be an effective carrier for bioformulations [50]. An important point to note is the potential biosecurity concerns with the use of *T. nordicum*-based biocontrol solutions. Another species, *T. afroharzianum*, has been reported to cause ear rot in maize [51] and is listed on the European and Mediterranean Plant Protection Organization alert list [52]. There is a need for careful evaluation of the potential unintended effects of *T. nordicum* before use in biocontrol solutions.

## 5. Conclusions

*T. nordicum* V1 can antagonize several oomycete plant pathogens and promote pepper growth, and can be used for biocontrol of seedling damping-off caused by *P. aphanidermatum*, and Phytophthora blight of pepper caused by *P. capsici*. The mechanisms underlying the antagonistic interactions between *T. nordicum* V1 and oomycetes, such as parasitic or predatory mechanisms, have not yet been demonstrated and require further investigation. Overall, *T. nordicum* V1 is a promising candidate to develop into a biocontrol agent for the biocontrol of oomycete-caused plant diseases. In the future, further taxonomic analysis is required to delineate the species boundaries of the *T. nordicum*/*T. nigricans* species complex.

## Figures and Tables

**Table 1:** Summary of the nucleotide sequence similarity of the *T. nordicum* V1 strain barcode sequences with those of the type strains for *T. nordicum*, *T. nigricans,* and *T. atroviride*.

Barcode	V1 Sequence ID (bp)	Trichoderma Strain	% Similarity (Sites)
		*T. nordicum* ACCC 39713 (WT13001) (bp)	
ITS	PQ489411.1 (571 bp)	NR_184883.1 (607 bp)	100% (571/571)
*tef1*	PQ510044.1 (551 bp)	MH287501.1 (1217 bp)	99% (526/530)
*rpb2*	PQ510043.1 (805 bp)	MH287502.1 (1175 bp)	100% (805/805)
		*T. nigricans* (T32781 = CGMCC40314) (bp)	
ITS	PQ489411.1 (571 bp)	OP341437.1 (533 bp)	100% (524/524)
*tef1*	PQ510044.1 (551 bp)	OP357974 (849 bp)	99% (482/484)
*rpb2*	PQ510043.1 (805 bp)	OP357959 (813 bp)	100% (614/614)
		*T. atroviride* CBS142.95 (bp)	
ITS	PQ489411.1 (571 bp)	MH862505.1 (633 bp)	99% (569/571)
*tef1*	PQ510044.1 (551 bp)	AF456891.1 (548 bp)	91% (449/491)
*rpb2*	PQ510043.1 (805 bp)	EU341801.1 (1009 bp)	97% (599/615)

## Data Availability

The data supporting the conclusions of the study are included in the main text or Supplementary Materials.

## Supplementary Materials

The following supporting information can be downloaded at https://www.mdpi.com/article/10.3390/jof12040292/s1: Figure S1. (A) Test of viability of *P. capsici* LT263 mycelia from parts overgrown by *T. nordicum* V1 isolate. Note that the growth of *P. capsici* plugs at 33 °C is slow, with growth mainly around the plug in the photo. (B) Summary of the numbers of plugs with growth after transfer from the indicated confrontation or single culture control plates. The viability tests were repeated two times with similar results; Figure S2. Time course of disease index and AUDPC for two repeat experiments (A,B) from control of *T. nordicum* on pepper blight caused by *P. capsici*. The error bars represent standard errors (*n* = 10 plants). *** = *p* < 0.001 from Dunn’s post hoc test after Kruskal–Wallis test in comparison to the disease index or AUDPC of seedlings inoculated with *P. capsici* LT263 and autoclaved *T. nordicum* spores; Figure S3. Re-isolation of *P. capsici* and *T. nordicum* from the pepper pot trial. Agarose gel of PCR reactions from gDNA from mycelia grown out from pepper root samples from indicated treatments from the pot trial, with information below the gel on the match of the sequencing of the ITS PCR products. (A) is from the first repeat and (B) is from the second repeat of the pepper pot-trial; Figure S4. Growth promotion effect of *T. nordicum* isolates on pepper from two repeat experiments (A,B). The error bars represent standard errors (*n* = 10 plants). * = *p* < 0.05, ns = non-significant from Dunnett’s post hoc test (after ANOVA) in comparison to the seedlings inoculated with autoclaved *T. nordicum* spores (-); Figure S5. Disease index of *P. myriotylum*-caused Pythium soft rot of ginger and biocontrol attempts using *T. nordicum* V1 isolate, whereby disease scores were recorded from 8 to 16 DAI with *P. myriotylum* SWQ7 where (A) shows the time course of disease index for all days recorded and AUDPC and (B) shows scores at 16 DAI. The pot trial was repeated twice with similar results. The error bars represent standard errors (*n* = 12). *** = *p* < 0.001, ns = non-significant from Dunn’s post hoc test after Kruskal–Wallis test in comparison to the disease index or AUDPC of seedlings inoculated with *P. myriotylum* SWQ7 and autoclaved *T. nordicum* spores. (C) Images of the ginger seedlings from the disease control pot trial. (D) Lack of growth promotion effect of *T. nordicum* V1 on ginger seedlings. The error bars represent standard error (*n* = 12). ns = non-significant from Dunnett’s post hoc test (after ANOVA) in comparison to the seedlings inoculated with autoclaved *T. nordicum* spores (-); Figure S6. Re-isolation of *P. myriotylum* and *T. nordicum* from the ginger pot trial. Agarose gel of PCR reactions from gDNA from mycelia grown out from ginger root samples from indicated treatments from the pot trial, with information below the gel on the match of the sequencing of the ITS PCR products; Table S1. Individual scores from the plate confrontation assay to screen the ability of *T. nordicum* V1 and V4 isolates to overgrow a range of oomycete plant pathogens. The following Excel nested IF function was used to categorize the *Trichoderma* colony coverage for Bell’s scale where LoT = the length of Trichoderma colony coverage: “=IF(LoT > 8.1,1,IF(LoT > 5.9,2,IF(LoT > 4.4,2.5,IF(LoT > 2.9,3,IF(LoT > 0.5,4,5)))))”.

## Abbreviations

The following abbreviations are used in this manuscript:

AUDPC	Area under the disease progress curve
AZCL	Azurine cross-linked
cV8	Clarified V8 juice
DAI	Days after inoculation
ITS	Internal transcribed spacer region
PDA	Potato dextrose agar
