# Supplementary figures and images for: Dual Biocontrol and Plant Growth-Promoting Effects of Trichoderma nordicum V1 Against Oomycete Plant Pathogens
Source: J Fungi (Basel). 2026 Apr 20;12(4):292. doi: 10.3390/jof12040292 (PMC13117944; doi:10.3390/jof12040292)

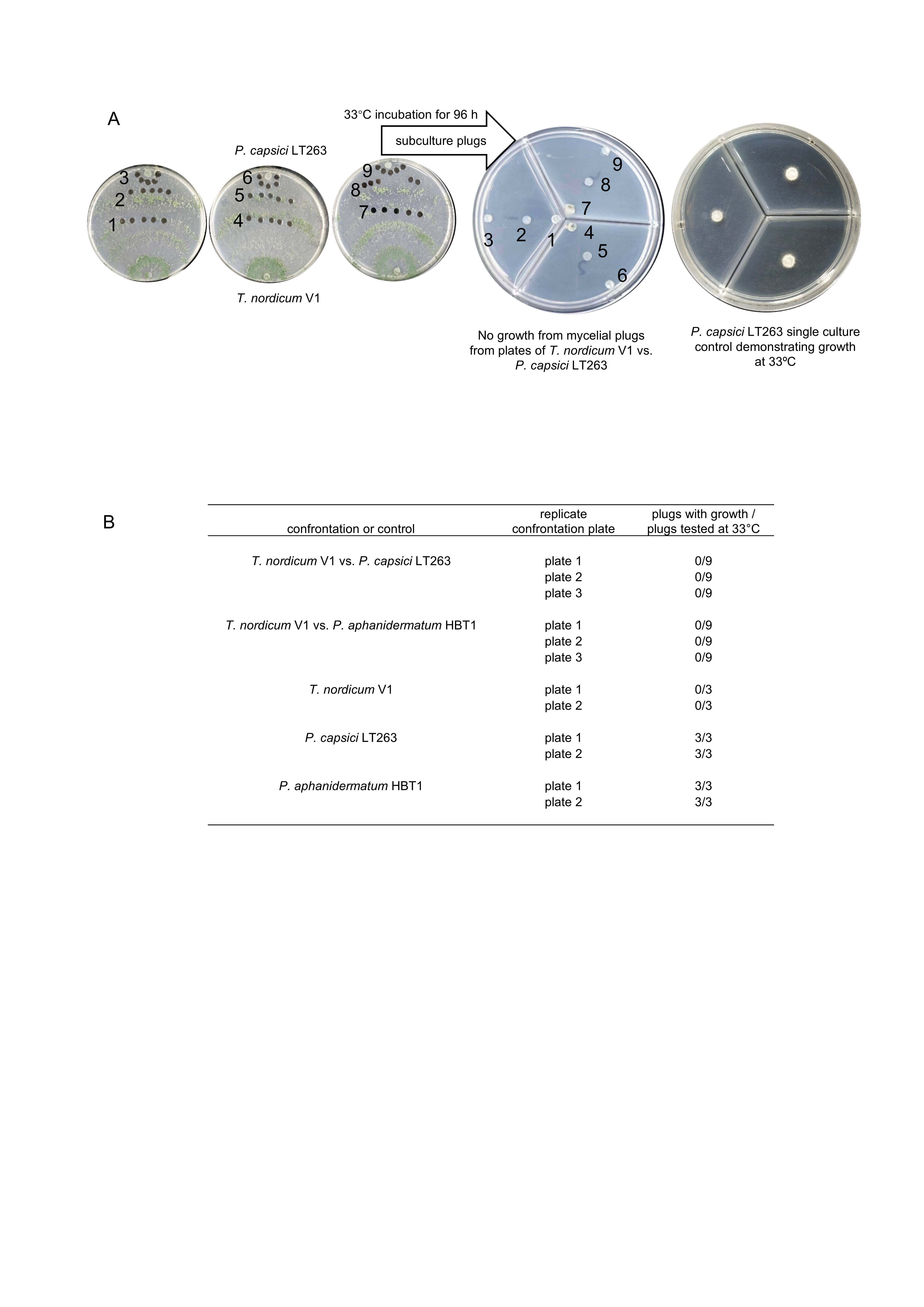

Supplement: Supplementary file 1 [file jof-12-00292-s001.zip › Figure_S1.tif]

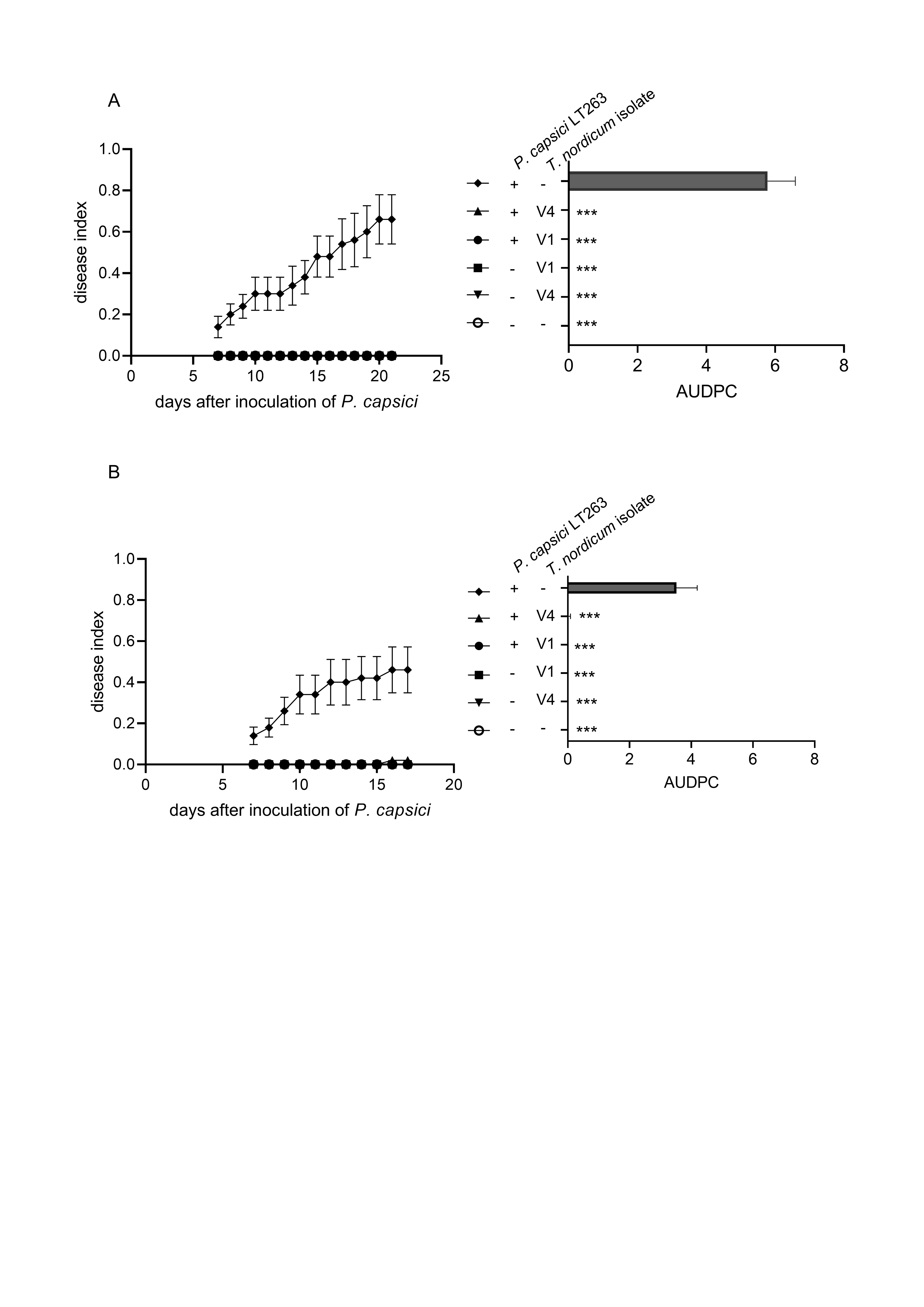

Supplement: Supplementary file 1 [file jof-12-00292-s001.zip › Figure_S2.tif]

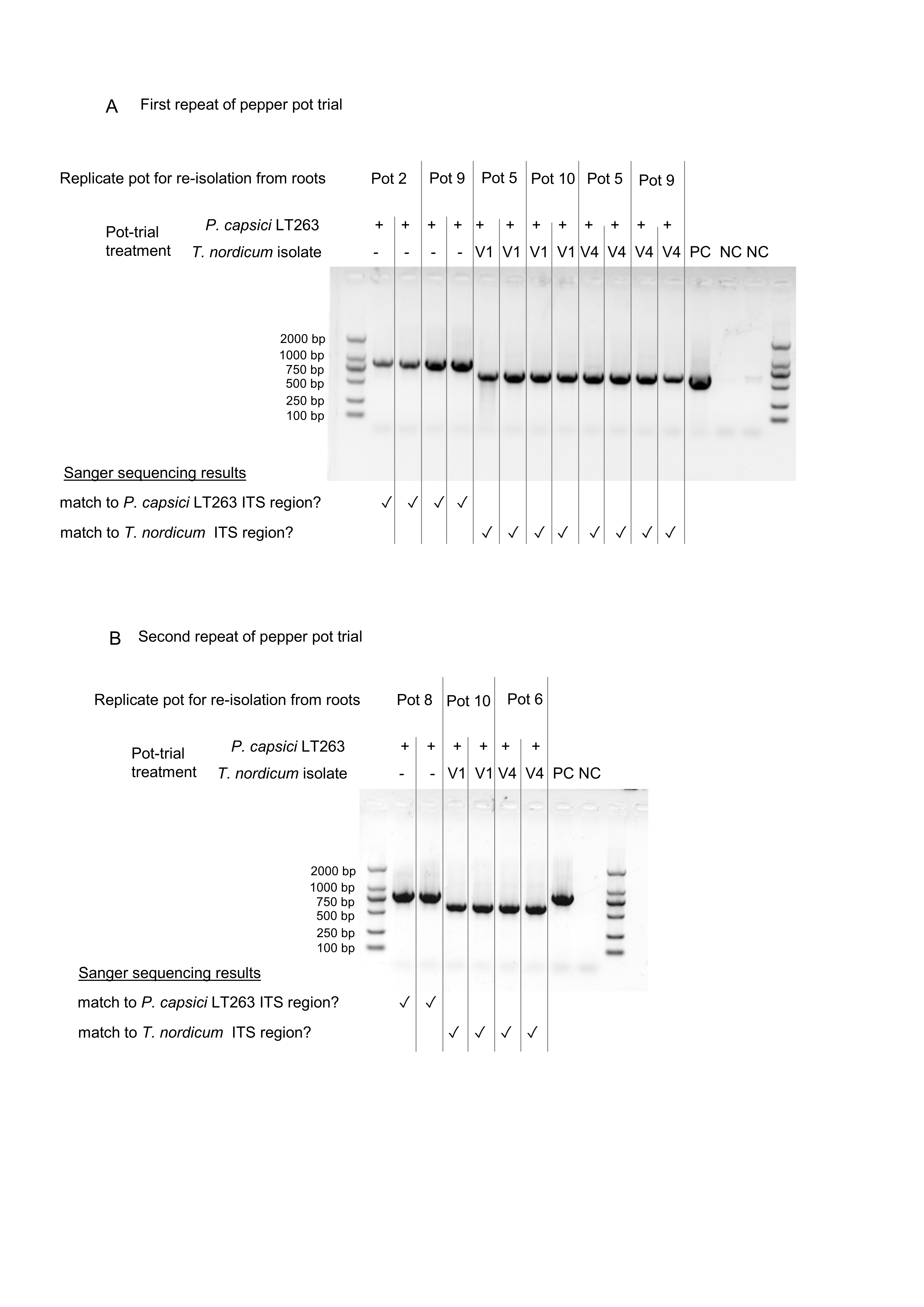

Supplement: Supplementary file 1 [file jof-12-00292-s001.zip › Figure_S3.tif]

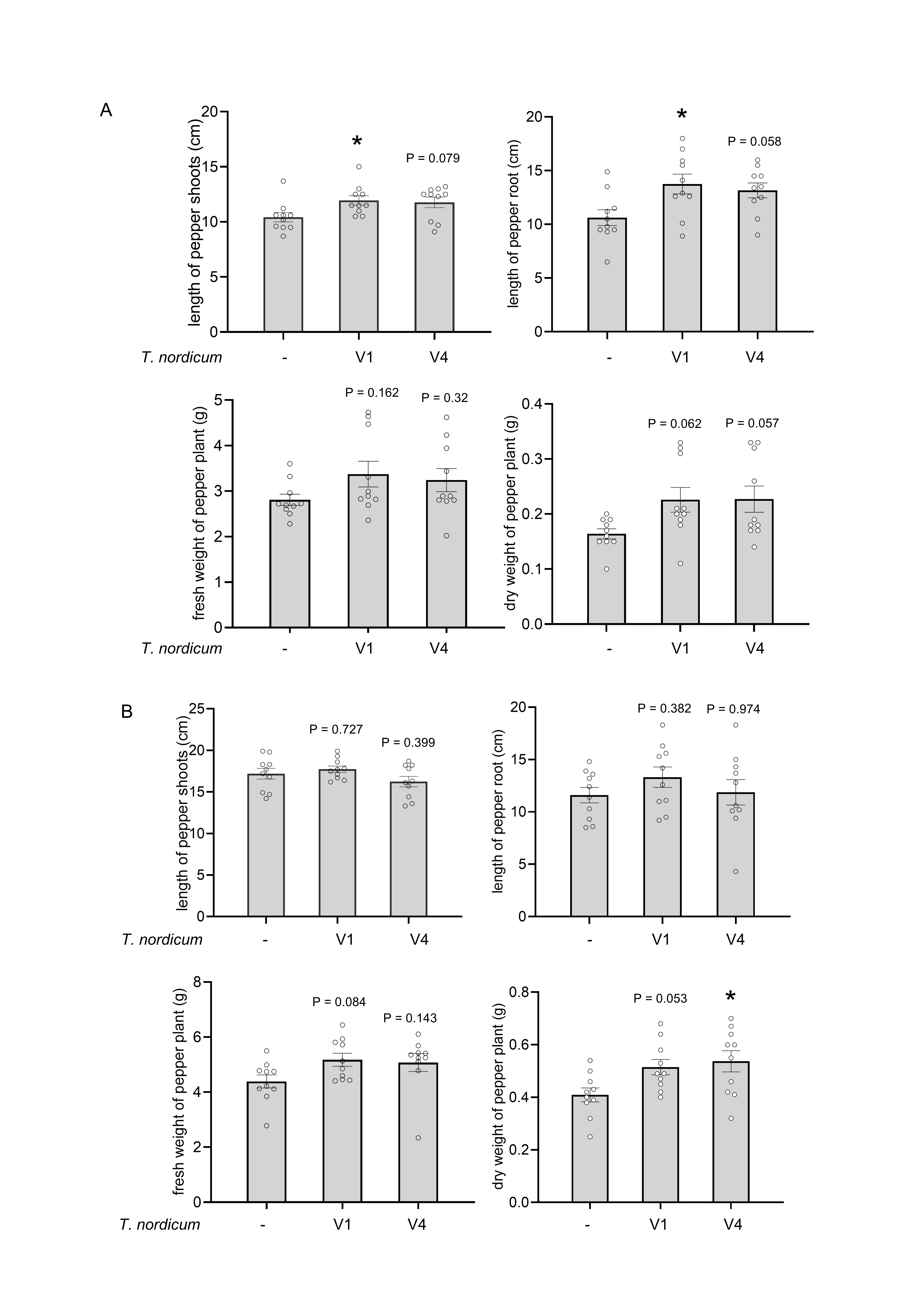

Supplement: Supplementary file 1 [file jof-12-00292-s001.zip › Figure_S4.tif]

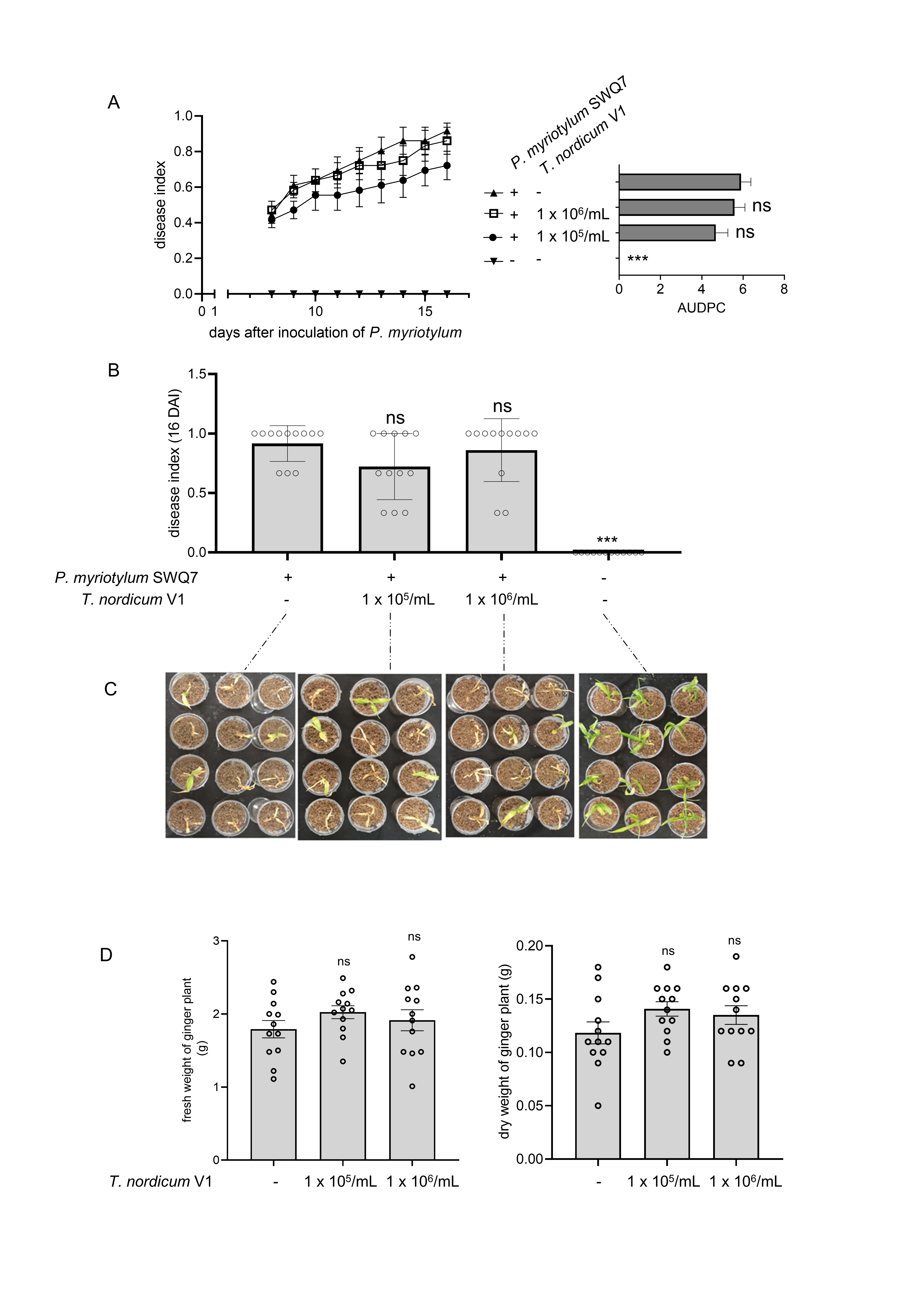

Supplement: Supplementary file 1 [file jof-12-00292-s001.zip › Figure_S5.tif]

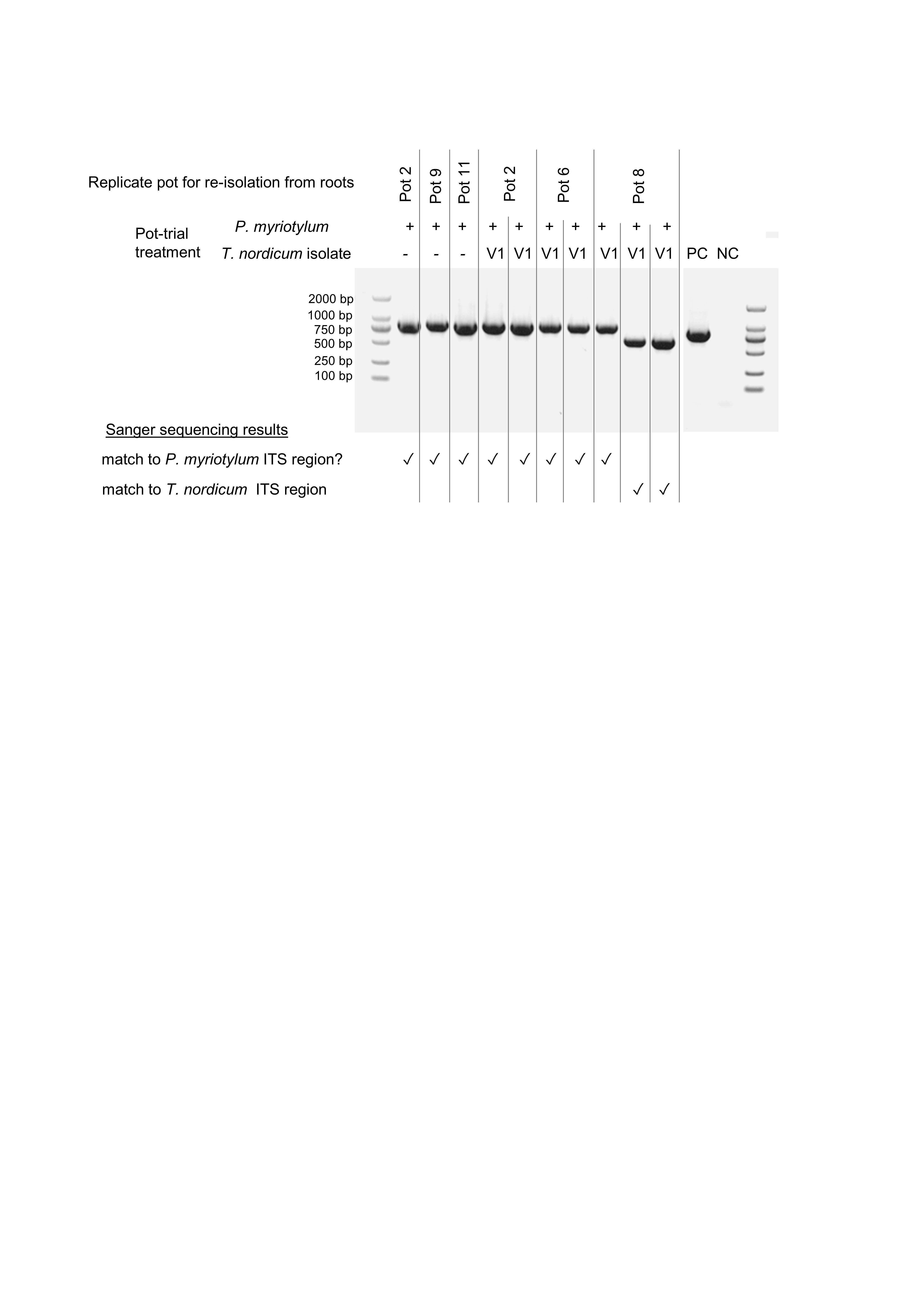

Supplement: Supplementary file 1 [file jof-12-00292-s001.zip › Figure_S6.tif]
